# CUL4A facilitates hepatocarcinogenesis by promoting cell cycle progression and epithelial-mesenchymal transition

**DOI:** 10.1038/srep17006

**Published:** 2015-11-23

**Authors:** Yingfang Pan, Bo Wang, Xiaoyun Yang, Fuxiang Bai, Qun Xu, Xueen Li, Lifen Gao, Chunhong Ma, Xiaohong Liang

**Affiliations:** 1 Key Laboratory for Experimental Teratology of Ministry of Education and Department of Immunology, Shandong University School of Medicine, Jinan, Shandong 250012, China; 2 Department of Gastroenterology, Qilu Hospital, Shandong University, Jinan 250012, PR China; 3 Department of Neurosurgery, Qilu Hospital, Shandong University, Jinan 250012, PR China; 4 Blood Group Reference & Research Laboratory, Shandong Blood Center, Jinan 250014, PR China

## Abstract

CUL4A, a member of the CULLIN family, functions as a scaffold protein for an E3 ubiquitin ligase. It was reported that the *CUL4A* gene showed amplification in some human primary hepatocellular carcinomas (HCC). However, the exact role of CUL4A in HCC remains unknown. Here, we aimed to investigate the expression and function of CUL4A in HCC development. Through immunohistochemistry study, we showed increased CUL4A expression in HCC tissues. Statistical analysis disclosed an inverse correlation between CUL4A expression and tumor differentiation grade, and patient survival, but a positive correlation with hepatocyte proliferation as well as lymphatic and venous invasion. CUL4A expression in HCC tissues was associated with HBeAg status in patients and upregulated by HBV in HCC cell lines. Further functional assay showed that CUL4A overexpression significantly promoted growth of H22 tumor homografts in BALB/c mice. Consistently, CUL4A knockdown inhibited the proliferation of established HCC cells, accompanied by S-phase reduction and Cyclin A and Cyclin B1 repression. Furthermore, CUL4A siRNA ameliorated the motility of HCC cell lines with altered expression of epithelial-mesenchymal transition (EMT)-associated molecules. Taken together, our findings indicate that CUL4A plays a pivotal role in HCC progression and may serve as a potential marker for clinical diagnosis and target for therapy.

Hepatocellular carcinoma (HCC), the overwhelming majority of liver cancers, is the sixth most common malignant tumor worldwide and the second most frequent mortal cancer, accounting for as many as half a million deaths annually^1^. Accumulated genetic and epigenetic alterations occurring in hepatocytes and the accompanied uncontrolled cell proliferation and death are essential for the initiation and progression of HCC^2^,^3^. Chromosomal abnormalities is the most common genetic changes in HCC and several hot chromosomal regions with frequent instability have been identified in HCC^4^. Among them, 13q34 amplification in 2 of 11 HCC cell lines involving 5 genes, including CUL4A, were also identified by FISH assay^5^. However, little is known about the exact role of CUL4A in HCC.

*Cullin 4A (CUL4A)*, a single-copy gene, encodes an 87-kDa protein belonging to the cullin family. CUL4A is highly expressed in testis and spleen while poorly expressed in lung, liver, thymus, small intestine, and muscular tissues^6^. As a scaffold protein, CUL4A binds with DNA damage binding protein 1 (DDB1) and ring of cullins (ROC1) and constitutes the ubiquitin ligase E3 complex, which mediates the ubiquitination and degradation of specific substrates. CUL4A E3 ubiquitin ligase has been known to be important for maintaining cellular physiology, including cell cycle, DNA replication, genomic stability, haematopoiesis, and spermatogenesis^7^. In recent years, the role of CUL4A in oncogenesis has attracted considerable attention. Amplification of *CUL4A* gene has been demonstrated in many kinds of tumors such as HCC^5^, squamous cell carcinomas^8^, and adrenocortical carcinoma^9^, etc. CUL4A overexpression was also found in epithelial ovarian tumours^10^, pituitary adenomas^11^, and breast cancer^12^. High CUL4A expression in node-negative breast cancers^13^, lung cancer^14^, and ovarian tumours^10^, promoted malignant transformation and correlated with shorter overall and disease-free survival. Recently, elevated CUL4A was found to be positively associated with distant metastasis of breast cancer by inducing epithelial-mesenchymal transition (EMT)^15^. More interestingly, it was reported that, besides tumor growth and metastasis, CUL4A was involved in conferring breast cancer cells to multiple drug resistance (MDR) by upregulating MDR1/P-gp expression^16^. On the other hand, CUL4A expression sensitized NSCLC cells to Erlotinib through transcriptionally regulating EGFR expression^14^. Thus, CUL4A, acting as an oncogene, significantly contributes to not only tumor initiation, but also progression. In addition, it might be valuable for prognosis and may serve as a target for drug development. However, the exact role and mechanism of CUL4A in HCC development remains largely unknown.

In the present work, we aimed to investigate the clinical and functional significance of CUL4A in HCC. High levels of CUL4A were found in HCC tissues and closely correlated with tumor differentiation grade and metastasis. Moreover, elevated CUL4A expression predicted poor overall survivals in patients with HCC. Ectopic CUL4A expression in homograft tumor promoted tumor growth in mice. Consistently, CUL4A knockdown led to reduce HCC cell growth, accompanied with a reduction of cells in S-phase. Also, CUL4A silencing suppressed the motility of HCC cells and reversed their EMT tendency. Our findings suggest that CUL4A promotes hepatocarcinogenesis, supporting the idea that CUL4A may become a potential prognostic marker and may serve as a therapeutic target.

## Results

### CUL4A is highly expressed in HCC tissues

**Figure 1:**
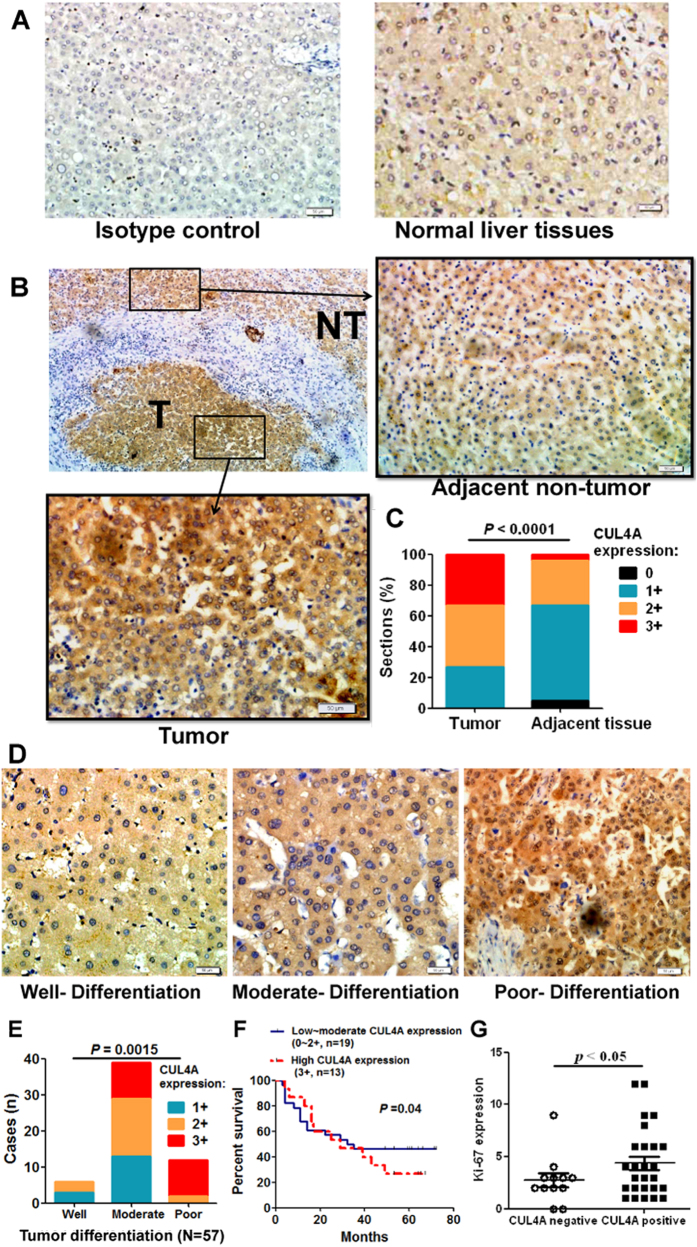
CUL4A is upregulated in HCC and closely related with the clinical parameters. (**A**) Immunohistochemical staining of CUL4A in normal liver tissues (right panel) and the corresponding isotype control (left panel). Original magnification 200×. (**B**) Immunohistochemical staining of CUL4A in HCC tissues and adjacent non-tumor tissues. T = Tumor. NT = adjacent non-tumor. Original magnification 200×. (**C**) Statistical data of CUL4A expression intensity in HCC tumor tissues and adjacent non-tumor tissues. (**D**,**E**) Immunohistochemical staining (**D**) and statistical analysis (**E**) of CUL4A in HCC tissues with well-, moderate-, and poor-differtiation. Original magnification 200×. (**F**) Survival curves of HCC patients with low~moderate versus high expression of CUL4A. (**G**) Statistical analysis of CUL4A expression and Ki-67 expression in HCC tissues.

Previous studies showed the amplification of CUL4A gene in HCC cell lines and clinical samples^5^. Here we examined CUL4A expression in normal liver tissues and HCC tissues by immunohistochemistry staining. Weak or intermediate immunoreactivity of CUL4A was detected in normal liver tissues (Fig. 1A). Similar CUL4A immunoreactivity was also observed in paratumor tissues (Fig. 1B, upper right panel). However, CUL4A expression was significantly increased in HCC tissues (Fig. 1B, lower left panel), when compared with paired adjacent non-tumor tissues (Fig. 1C). Moreover, high level of CUL4A was positively associated with tumor pathological grade (Fig. 1D,E). CUL4A expression in poorly-differentiated tumors was significantly higher than that in well-differentiated or moderately-differentiated tumor tissues (*P* = 0.0006, Table 1). In addition, increased CUL4A staining correlated with reduced overall survival times of patients (Fig. 1F). Consistently, levels of hepatocyte proliferation (Ki-67 as the marker) were significantly higher in CUL4A-positive HCC tissues (staining score: 5-12) than CUL4A-negative tissues (staining score: 0–4) (Fig. 1G). Moreover, the increase of CUL4A expression was more significant in tumors with diameters more than 5 cm and in tumors with lymphatic and venous invasion (Table 1). Collectively, our data demonstrate that increased CUL4A expression correlates with the progression of HCC and CUL4A might be involved in promoting tumor growth and metastasis.

### HBV and its viral proteins upregulate CUL4A expression

**Figure 2:**
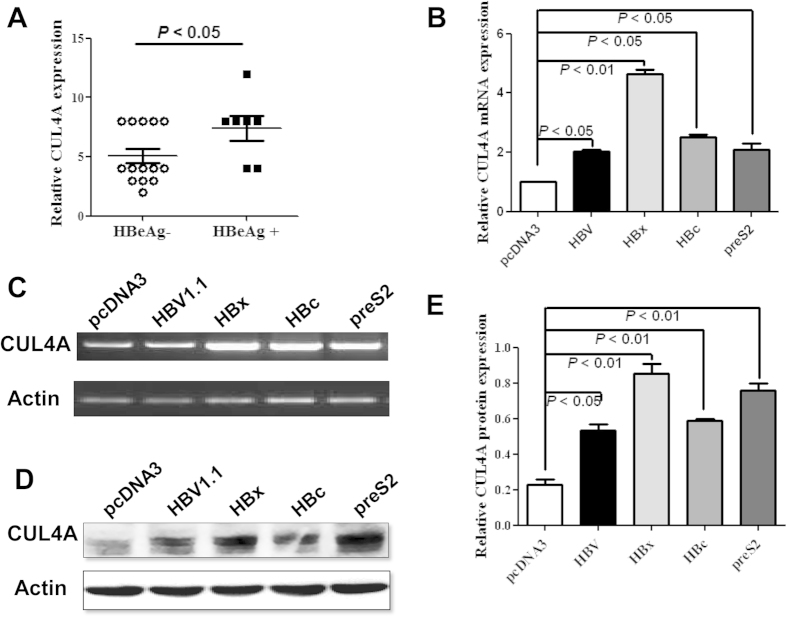
Upregulation of CUL4A by HBV and its viral proteins. (**A**) Statistical analysis of CUL4A expression in patients with HBeAg negative or positive HCC. (**B–E**) BEL7402 cells were transfected with pcDNA3-HBV1.1, pcDNA3-HBx, pcDNA3-HBc, pcDNA3-preS2, or control pcDNA3 plasmid. Forty-eight hours later, real-time (**B**) or semi-quantitative (**C**) RT-PCR and Western blot (**D**) was performed. The relative CUL4A protein level, expressed as CUL4A/β-actin ratio, was determined by densitometry with ImageJ software (**E**).

Based on our data, CUL4A expression was increased in about 71% HCC tissues, which was significantly higher than the frequency of the CUL4A gene amplification in HCC tissues (3/51) reported before^5^. The difference suggested that there were other factors contributing to the upregulation of CUL4A in HCC tissues. It is well-known that HBV infection is a major etiological factor for HCC development^17^. In our study cohort, almost all patients were serum HBsAg positive. Considering that HBeAg could be an indicator of HBV replication^18^, we analyzed the correlation of serum HBeAg status in patients with CUL4A expression. As shown in Fig. 2A, serum HBeAg-positive patients had a higher CUL4A expression than HBeAg-negative patients. Moreover, both full-length HBV genome and HBV encoded proteins (HBx, preS2, and HBc) enhanced CUL4A mRNA expression in HCC cell lines (Fig. 2B,C). Consistently, the expression of CUL4A protein was also significantly upregulated by full-length HBV genome and viral proteins HBx and preS2 (Fig. 2D,E). Taken together, these data indicate that HBV infection contributes to the upregulation of CUL4A in HCC patients.

### CUL4A promotes HCC growth both *in vivo* and *in vitro*

**Figure 3:**
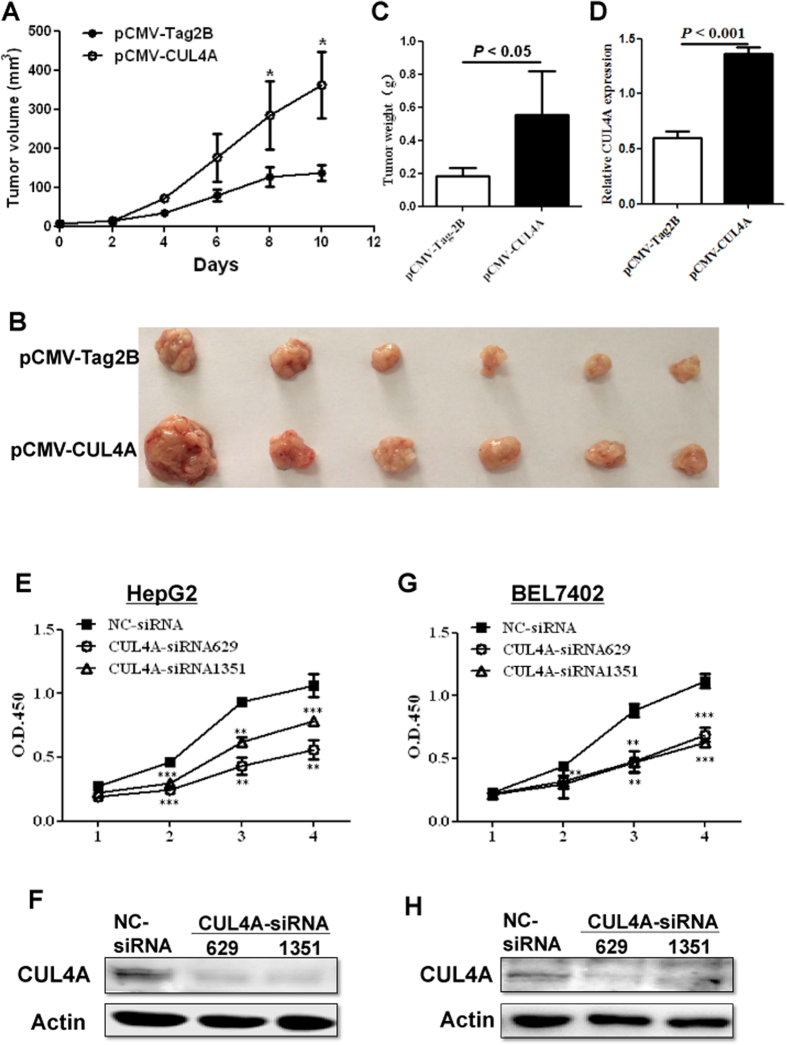
CUL4A modulates the growth of HCC both *in vivo* (A–D) and *in vitro* (E–H). (**A**–**D**) BALB/c mice were subcutaneously implanted with murine hepatoma cells H22 and pCMV-Tag2B or pCMV-CUL4A was intratumorally injected. (**A**) Homograft tumor growth determined over a 10 day period. (Mean ± SD, n = 6); *p < 0.05. (**B**) Images of tumors from each group are shown. (**C**) The tumor weight at the time of sacrifice. Summary data of each group is shown (n = 6). (**D**) CUL4A overexpression efficiency was confirmed by RT-PCR. (**E**–**H**) Proliferation of HepG2 and BEL7402 cells transfected with negative control siRNA (NC-siRNA) or CUL4A siRNAs. The growth curve of HepG2 (**E**) or BEL7402 (**G**) cells was determined by CCK-8 assay during a 4-day period and data shown is Mean ± SD from three experiments. The knockdown efficiency of CUL4A-siRNAs in HepG2 (**F**) or BEL7402 (**H**) cells was confirmed by Western blot.

To further investigate the role of CUL4A in HCC development, we measured the growth of H22 tumor homografts in BALB/c mice where CUL4A expression was modulated. After the tumor diameter reached 0.5 cm, pCMV-Tag2B or pCMV-CUL4A was injected intratumorally. Tumor growth curve showed that pCMV-CUL4A significantly promoted the growth of H22 tumor homografts over the period of the experiment (Fig. 3A,B). Consistently, pCMV-CUL4A injected tumors at the time of sacrifice weighed more than pCMV-Tag2B injected group (Fig. 3C) and had higher expression of CUL4A (Fig. 3D). Collectively, these results suggest that CUL4A promotes the growth of HCC.

The influence of CUL4A on HCC growth was further validated by measuring the growth of HCC cell lines *in vitro*. The HCC cell lines HepG2 and BEL7402, with high endogenous level of CUL4A expression, were transfected with two siRNAs specifically targeting CUL4A. The proliferation of both cell lines was significantly inhibited by CUL4A siRNA over a four-day period (Fig. 3E,G). The knockdown efficiency of CUL4A in both cell lines was confirmed by Western blot (Fig. 3F,H). Taken together, both *in vivo* and *in vitro* studies indicate that CUL4A promotes cell growth and support the idea that CUL4A functions as an oncogene in HCC development.

### CUL4A knockdown correlates with S-phase reduction and Cyclin A and Cyclin B1 repression

**Figure 4:**
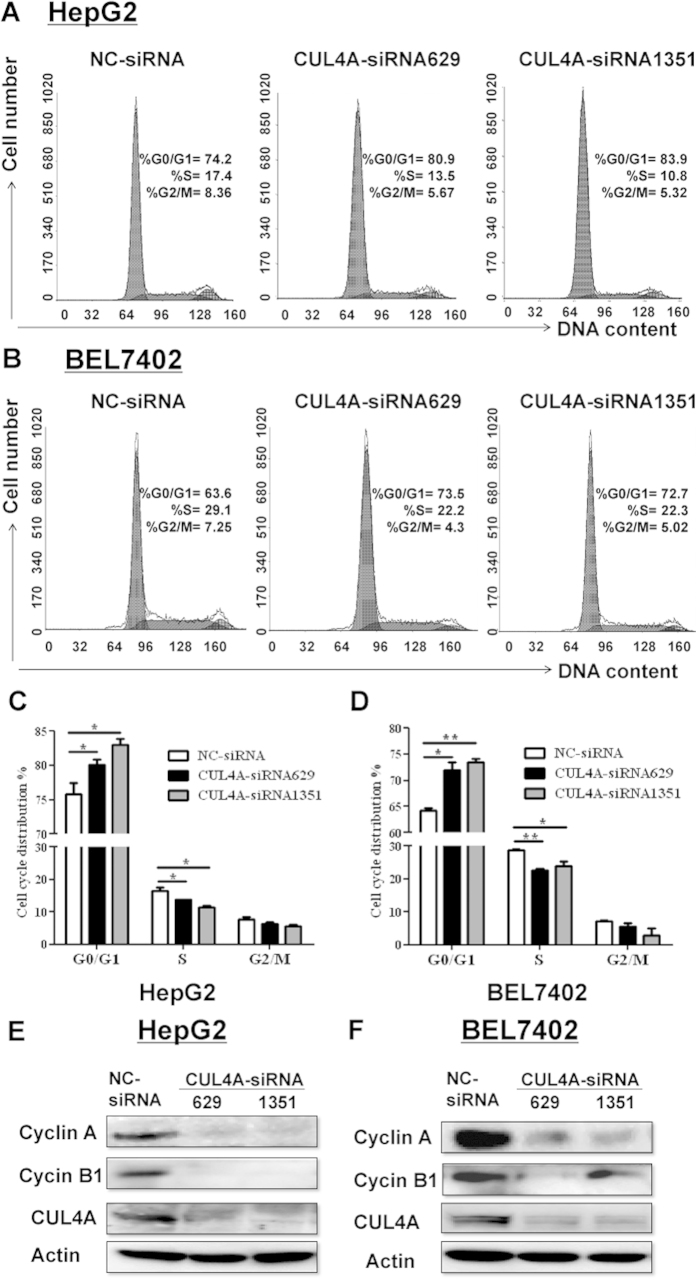
Silencing CUL4B coincides with S phase reduction and Cyclin A and Cyclin B1 repression. (**A**) HepG2 or (**B**) BEL7402cells transfected with NC-siRNA or CUL4A-siRNAs were analyzed by PI staining and flow cytometry. (**C**,**D**) Statistical analysis of cell cycle distribution of each group from experiments (**A** or **B**). (**E**,**F**) Western blot was performed to monitor the expression of CUL4A, Cyclin A and Cyclin B1 in the HCC cell lines described in (**A**,**B**).

To explore the mechanisms for CUL4A promoting the proliferation of HCC cells, cell cycle profile in two HCC cell lines, where CUL4A expression was knocked down, was analyzed by flow cytometry. Transfection of CUL4A siRNA coincided with the decreased percentage of S phase cells in both HepG2 (Fig. 4A,C) and BEL7402 cells (Fig. 4B,D). In addition, the expression of potential cell cycle regulators was examined. Western blot results showed that CUL4A knockdown accompanied with the downregulated expression of Cyclin A and Cyclin B1 in both HepG2 (Fig. 4E) and BEL7402 cells (Fig. 4F). Collectively, these results indicate that CUL4A expression correlates with the deregulated cell cycle progression and the altered expression of cell cycle-related proteins in HCC cell lines.

### CUL4A knockdown inhibited HCC cell migration and invasion by reversing EMT tendency

**Figure 5:**
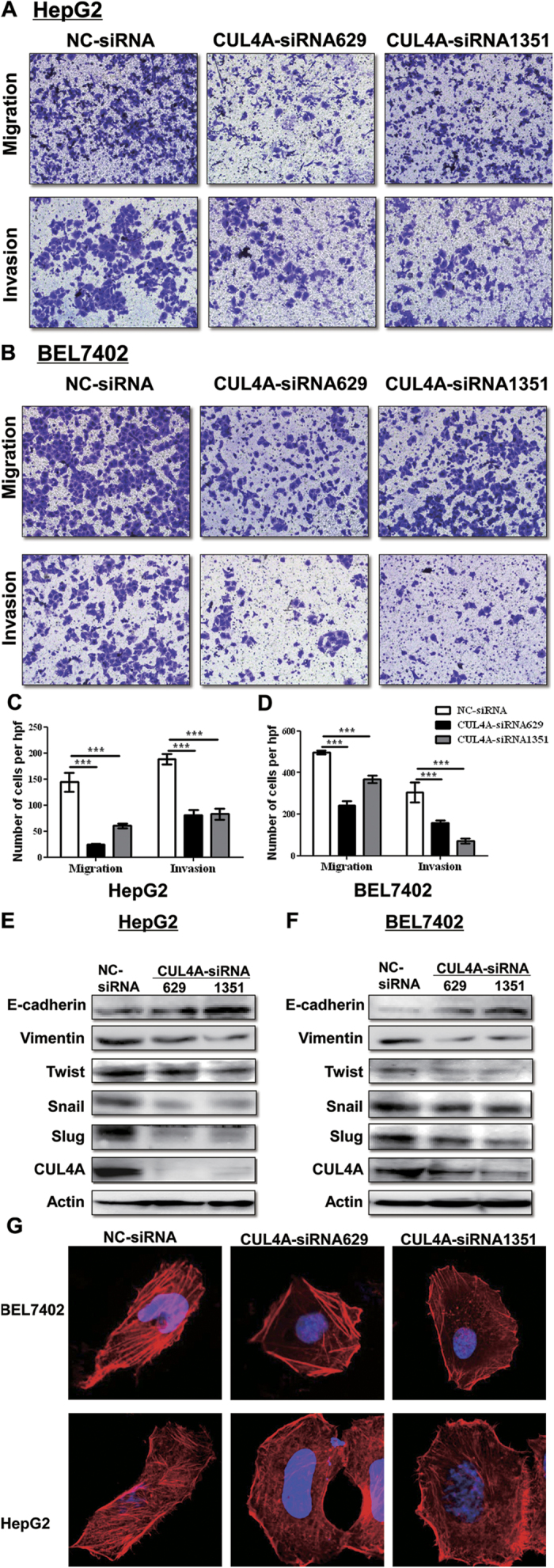
Interference of CUL4A attenuated HCC cell motility and the EMT tendency. (**A**,**C**) HepG2 cells transfected with NC-siRNA or CUL4A-siRNAs were applied for Transwell assay. Representative images for migration and invasion assay were shown (**A**). Statistical analysis of each group is shown as Mean ± SD from three experiments (**C**). (**B**,**D**) Migration and invasion assay were also performed in BEL7402 cells as described in 5A and 5C and the representative images (**B**) and the statistical data were shown (**D**). (**E**,**F**) Western blot to determine the expression of CUL4A, E-Cadherin, Vimentin, Snail, Slug, and Twist in HepG2 (**E**) or BEL7402 (**F**) cells transfected with NC-siRNA or CUL4A-siRNA. (**G**) The actin filaments in BEL7402 cells (upper panel) or HepG2 cells (lower panel) transfected with NC-siRNA or CUL4A-siRNAs were stained Tetramethylrhodamine (TRITC)-conjugated phalloidin and observed under laser confocal microscopy.

Our analysis with clinical samples suggested that increased CUL4A expression correlated with lymphatic and venous invasion of HCC tissues (Table 1). To further evaluate the role of CUL4A in controlling cell motility, Transwell assay combined with Matrigel analysis were performed in both HCC cell lines transfected with CUL4A siRNA. As shown in Fig. 5A,C, silencing CUL4A dramatically weakened the migratory and invasive capacity of HepG2 cells. Similar results were also obtained in BEL7402 cells (Fig. 5B,D). Thus, these results indicate that CUL4A enhances the migration and invasion of HCC cell lines.

Considering the importance of EMT in the progression of HCC^19^, we determined the expression pattern of epithelial and mesenchymal markers on HCC cell lines transfected with CUL4A-siRNAs or control NC-siRNA. Western blot results showed that silencing CUL4A increased the level of epithelial markers (E-Cadherin), but decreased the expression of mesenchymal markers (Vimentin) as well as EMT-promoting transcription factors including snail, slug and twist (Fig. 5E,F). Also, we detected actin fiber formation in CUL4A knockdown or control cells by immunofluorescence. As shown in Fig. 5G, compared with control cells, CUL4A silencing BEL7402 and HepG2 cells showed reduced actin fiber formation. Taken together, these results support the idea that CUL4A promotes the metastatic property of HCC cells by inducing EMT.

## Discussion

CUL4A was described as an oncogene in many kinds of tumors^6^,^7^,^8^,^9^,^10^,^11^,^12^,^13^,^14^. Amplification of *CUL4A* gene in several HCC cell lines and clinical HCC samples^5^ indicated its potential involvement in the pathogenesis of HCC. However, the exact role of CUL4A in the development and progression of HCC remains unknown. In the present study, we for the first time reported that CUL4A protein expression was significantly upregulated in HCC tissues. More importantly, aberrant expression of CUL4A was more pronounced in poorly-differentiated HCC tissues and tumors more than 5 cm in diameter, indicating that CUL4A participates in the progression of HCC.

One important question is how CUL4A expression is upregulated in HCC. It is well-accepted that complicated genetic or epigenetic alterations in hepatocytes are involved in finely tuning the expression of oncogenes and tumor suppressor genes, which in turn transform the normal hepatocytes and lead to hepatocarconogenesis^4^. Although it was reported previously that *CUL4A* gene amplification occurred in several HCC cell lines and tumor tissues^5^, it could not fully explain the overexpression of CUL4A protein occurring in most of HCC cases, as indicated in our immunohistochemistry staining assay. Thus, there might be other factors contributing to the upregulation of CUL4A in HCC development. HBV infection is the most important cause of HCC worldwide. It is well-established that HBV and its encoded proteins greatly contribute to these processes by regulating the expression of multiple tumor-related genes, such as human telomerase reverse transcriptase (TERT), β-catenin, and Snail, etc^20^. In our study cohort, ~90% of HCC cases are HBsAg positive. Interestingly, the statistical analysis showed the positive correlation between the presence of serum HBeAg and CUL4A expression in HCC tissues, indicating the regulatory role of HBV on CUL4A expression. Moreover, transfection of full-length HBV genome and viral gene fragment, HBx, HBc and preS2, had a significant induction of CUL4A expression. All these data suggest that HBV might be closely associated with the upregulation of CUL4A in HCC. However, the exact mechanisms for this role of HBV needs to be further investigated.

Consistent with upregulation of CUL4A in poorly-differentiated and large-size tumors, both *in vitro* and *in vivo* assay demonstrated that CUL4A had the ability to promote the proliferation of HCC cells. This raises another important question, how CUL4A promotes the growth of HCC cell lines? It is well-known that CUL4A induces cell cycle progression in normal cells by regulating the expression of cyclin-dependent kinase (CDK) inhibitors (CDIs), such as p21^CIP1/WAF1^, p27^KIP1^, and p16^INK4a^ 7, which is important for the maintenance of normal cell proliferation and survival. However, in tumor cells, overexpression of CUL4A instead deregulates cell cycle and then results in the uncontrolled proliferation of malignant cells^21^,^22^. Here, we found that silencing CUL4A correlated with the reduced S-phase cells and downregulation of Cyclin A and Cyclin B1 (Fig. 4), while the expression of other cell cycle-related proteins remained unchanged (our unpublished data). Cyclin A, binding with CDK2, is responsible for controlling S phase progression and G2/M transition^23^. Cyclin B1 associates with CDK1 and promotes the process of mitotic cell division^24^. The deregulated expression of Cyclin A and Cyclin B1 was involved in reinforcing the malignant growth of HCC^25^,^26^. Detailed mechanisms for CUL4A modulation on Cyclin A and Cyclin B1 expression requires further study.

The correlation between high CUL4A expression and lymphatic and venous invasions suggests a potential role of CUL4A in metastasis of HCC tumors. The *in vitro* transwell assay also supports our hypothesis. Furthermore, Western blot results showed that CUL4A knockdown upregulated the expression of epithelial marker (E-cadherin), but downregulated the steady-state level of mesenchymal marker (Vimentin), and EMT-associated transcription factors. Also, immunofluorescence study indicated that CUL4A silencing reduced the actin fiber formation. EMT has been demonstrated to play an important role in the migration, invasiveness, metastasis and chemoresistance and has been highlighted as potential therapeutic target of HCC^27^,^28^. Our data indicate that CUL4A might accelerate the progression of HCC by promoting EMT tendency, although the exact mechanisms by which CUL4A affects EMT progression needs to be further investigation.

In conclusion, our study indicates that CUL4A controls cell proliferation and Cyclin A and Cyclin B1 are upregulated. These data provide new evidence for the tumor-promoting roles of CUL4A in liver cancer. Also, we identify the roles of CUL4A in the migration and invasion of HCC cell lines, which might be associated with EMT progression. Further studies are required to understand the detailed mechanisms by which CUL4A promotes cell cycle and EMT progression in HCC.

## Materials and Methods

### Clinical HCC samples

Fifty-seven tumor tissues and the corresponding adjacent non-tumor tissues were collected from patients with primary HCC who underwent surgical resection between Jan. 2012 and Mar. 2013 at Shandong University Qilu Hospital and Shandong Provincial Hospital. Cell differentiation-based HCC tumor grading was determined by the experienced pathologists according to the criteria described by Edmondson and Steiner^29^. HBV antigens in serum of patients were determined by ELISA. Patients with evidence of HCV or HIV infection, consumed excessive alcohol or received chemotherapy prior to surgery were excluded. Written informed consent was obtained from all patients, and the study was approved by the Shandong University Medical Ethics Committee in accordance with the Declaration of Helsinki.

### Immunohistochemistry staining

Immunohistochemistry was performed according to standard protocols using the following antibodies: anti-CUL4A (ab34897, Abcam) and anti-Ki-67 (ab15580; Abcam). Ten fields of ~1000 cells from each tumor and non-tumor section was independently counted by three pathologists. The level of CUL4A expression was reported according to the German semi-quantitative scoring system^30^,^31^. Briefly, CUL4A expression in each sample was scored according to staining intensity (no staining = 0; weak staining = 1; moderate staining = 2; strong staining = 3) and the number of stained cells (0% = 0; 1–25% = 1; 26–50% = 2; 51–75% = 3; 76–100% = 4). The final scores of immunoreactive were determined by multiplying the staining intensity by the number of stained cells, with minimum and maximum scores of 0 and 12, respectively^32^. Different staining grade was ranked according to the final staining scores: 0, total score = 1; 1+, total score = 1–4; 2+, total score = 5–8; 3+, total score = 9–12. The levels of hepatocyte proliferation were shown by the staining intensity of Ki-67, which was determined as described for CUL4A staining.

### Cell lines, plasmids and siRNAs

The human HCC cell lines HepG2 and BEL7402 were purchased from the Shanghai Cell Collection, Chinese Academy of Sciences. HepG2 cells were cultured in DMEM, and BEL7402 cells were maintained in RPMI1640. All culture media were supplements with 10% fetal bovine serum (FBS), 50 U/ml penicillin, and 50 U/ml streptomycin (Invitrogen, Beijing, China). Murine liver cancer cell line H22 was maintained in our laboratory and was passaged weekly in ascites fluid of BALB/C mice^33^.

The plasmid pCMV-CUL4A was constructed by cloning mouse full-length CUL4A gene fragment into pCMV-Tag2B as described previously^34^. The plasmids containing 1.1-fold genome of HBV, HBx, HBc, or preS2 gene fragment were constructed on the backbone of the mammalian expression vector pcDNA3 as described previously^35^,^36^,^37^.

The specific siRNAs against human CUL4A (CUL4A-siRNA629: 5’-CCAUCUGGGAUAUGGGAUUTT-3′; CUL4A-siRNA1351: 5′-GCAAAGCAUGUGGAUUCAATT-3′) and a scrambled control siRNA (NC-siRNA: 5′-CUCCGAACGUGUCACGUTT-3′) were synthesized by GenePharma.

### *In vivo* tumor homograft assay

Six to eight week-old, male BALB/c mice were purchased from the Animal center of Shandong University (Jinan, China) and housed in ventilated microisolator cages in a pathogen-free facility. Individual mice were implanted subcutaneously with 2 × 10^5^ H22 murine liver cancer cells suspended in 100 μl phosphate buffer solution. When the diameter of tumors reached 5 mm, the mice were randomly divided into two groups (6 mice for each group) and injected intratumorally with pCMV-Tag2B or pCMV-CUL4A (20 ug in 100 ul saline) every three days for a total 3 injections. The tumor sizes in each mouse were monitored using a vernier caliper every other day until three days after the last injection. Then, the mice were sacrificed and the tumors were isolated and weighed. CUL4A expression in tumors was performed by RT-PCR. All experiment protocols were carried out in accordance with the guidelines, which were approved by the Shandong University Animal Care Committee.

### Cell proliferation assay

The impact of CUL4A expression on the proliferation of HCC cells was determined by cell proliferation assay as described previously^38^. Briefly, BEL7402 or HepG2 cells at 10^4^ cells/well were cultured in 96-well plates overnight and transfected with CUL4A-siRNA or NC-siRNA using LipofectamineTM 2000 (Invitrogen), followed by incubation for four days. The proliferation of HCC was determined longitudinally using the CCK-8 kit (Beyotime, Shanghai, China) and measuring the absorbance at A450 in a microreader (Bio-Rad, Tokyo, Japan). CUL4A expression was quantified by Western blot.

### Cell cycle analysis

BEL7402 or HepG2 cells were collected 48 hrs after transfection with CUL4A-siRNA or NC-siRNA, stained with propidium iodide (PI, Sigma) and then assayed using a Beckman Coulter Flow Cytometer (Fullerton).

### *In vitro* invasion and migration assay

The cell migration assay was performed by using Transwell (Corning Life Sciences, Acton, MA). Around 3 × 10^4^ transfected cells (HepG2 or BEL7402) suspended in serum-free media were seeded on the upper side of the transwell chamber, which was transferred to a 24-well containing full culture medium containing 10% FBS. Twelve hours after incubation at 37 °C, cells on the upper side of the chamber were removed by wiping the top of the membrane with cotton swabs and the cells migrated to the lower side of the transwell membrane were fixed and stained in 0.1% crystal violet. Cells were counted from ~10 microscope fields and the mean value was shown. For cell invasion assay, transfected cells were seeded into matrigel (BD Biosciences, San Jose, CA) -coated transwell chamber and were incubated for 24 hours before staining. Each experiment was performed in replicate inserts and the mean value expressed from three independent experiments.

### Immunofluorescence staining and microscopy

Cells were seeded on cover slips, fixed with 2% paraformaldehyde, permeabilized with 0.2% Triton X-100 in phosphate-buffered saline (PBS) and then stained with Tetramethylrhodamine (TRITC)-conjugated phalloidin (Sigma-Aldrich, Louis, USA) For nuclear staining, cells were stained with 4’,6-diamidino-2-phenylindole (DAPI) (Beyotime). Images were taken using a confocal laser microscope (Carl Zeiss, LSM780, Oberkochen, Germany).

### Western Blot

BEL7402 or HepG2 cells, transfected with CUL4B-siRNA or NC-siRNA for 48 h, were harvested and total cell lysates were prepared, as previously described^35^. The cell lysates (20 μg/lane) were separated by sodium dodecyl sulfate polyacrylamide gel electrophoresis (SDS-PAGE) and transferred on polyvinylidene difluoride (PVDF) membranes. After being blocked with 5% fat-free dry milk, the membranes were incubated with antibodies against CUL4A (ab34897, Abcam), Cyclin A (4656, Cell Signal Technology), Cyclin B1 (ab32053, Abcam), Twist (ab50581, Abcam), Slug (9585, Cell Signaling), Snail (3879,Cell Signaling), Vimentin (BS1491, Bioworld), E-cadherin (3195, Cell Signaling), and β-actin (A5316, Sigma, St. Louis, USA), respectively. The bound antibodies were detected with HRP-conjugated second antibodies and visualized using Western Lightning Chemilluminescence Reagent (Amersham Biosciences, Piscataway, USA).

### Semi-quantitative and real-time RT-PCR analysis

BEL7402 cells were transfected with pcDNA3-HBV1.1, pcDNA3-HBx, pcDNA3-HBc, pcDNA3-preS2, or pcDNA3. Total RNA from cells transfected for 48 h or H22 tumor tissues, was extracted using TRIzol (TaKaRa, Tokyo, Japan), according to the manufacturers’ protocol. The total RNA was reversely transcribed into cDNA, and the relative levels of CUL4A mRNA transcripts to the internal control β-actin were determined by semi-quantitative or real-time PCR using the specific primers.

### Statistical Analysis

GraphPad Prism (GraphPad Software, San Diego, CA) was used for data analysis. The differences between groups were determined by the Student t-test or Mann-Whitney U test. The statistical correlation between the clinical parameters of HCC and the CUL4A staining levels in tissue sections was analyzed by the Chi-square test. Survival differences were analyzed using the log-rank test. In these analyses, p values < 0.05 were considered to be statistically significant.

## Additional Information

**How to cite this article**: Pan, Y. *et al.* CUL4A facilitates hepatocarcinogenesis by promoting cell cycle progression and epithelial-mesenchymal transition. *Sci. Rep.* **5**, 17006; doi: 10.1038/srep17006 (2015).

## Figures and Tables

**Table 1:** CUL4A expression, clinical and histologic features in 57 patients with HCC.

Clinical characteristic	No. of cases	CUL4A expression		
		Positive (5–12)	Negative (0–4)	Median ± SD (range)
Gender				
Female	5	4	1	8 ± 3.35 (4–12)
Male	52	39	13	8 ± 3.14(4–12)
*P* value		*0.3771*^*a*^		*0.3475*^*b*^
Age(year)				
≤50	16	10	6	8 ± 3.23 (4–12)
>50	41	32	9	8 ± 3.14 (4–12)
*P* value		*0.1058*^*a*^		*0.1197*^*b*^
Tumor diameter				
≤5cm	28	19	9	8 ± 2.75(0–16)
>5cm	29	24	5	9 ± 3.09(0–16)
*P* value		*0.0957*^*a*^		*0.0064*^*b*^
Differentiation grade				
well	6	3	3	5 ± 1.97(4–8)
moderate	39	26	13	8 ± 3.14(4–12)
poor	12	12	0	12 ± 1.75(8–12)
*P* value		*0.036*^*a*^		*0.0006*^*b*^
Lymphatic and Venous invasion				
absent	43	28	15	8 ± 3.25(4–12)
present	14	14	0	8.5 ± 2.21(4–12)
*P* value		*0.0040*^*a*^		*0.0058*^*b*^

*p*^*a*^ values were obtained from the Fisher’s exact test.

*p*^*b*^ values were obtained from the Student t-test.

## References

[b1] Sherman M. Hepatocellular carcinoma: epidemiology, surveillance, and diagnosis. Semin Liver Dis 30, 3–16, 10.1055/s-0030-1247128 (2010). 20175029

[b2] Aravalli R. N., Steer C. J. & Cressman E. N. Molecular mechanisms of hepatocellular carcinoma. Hepatology 48, 2047–2063, 10.1002/hep.22580 (2008). 19003900

[b3] Nishida N. & Goel A. Genetic and epigenetic signatures in human hepatocellular carcinoma: a systematic review. Curr Genomics 12, 130–137, 10.2174/138920211795564359 (2011). 21966251 PMC3129047

[b4] Liu M., Jiang L. & Guan X. Y. The genetic and epigenetic alterations in human hepatocellular carcinoma: a recent update. Protein Cell 5, 673–691, 10.1007/s13238-014-0065-9 (2014). 24916440 PMC4145080

[b5] Yasui K. *et al.* TFDP1, CUL4A, and CDC16 identified as targets for amplification at 13q34 in hepatocellular carcinomas. Hepatology 35, 1476–1484, 10.1053/jhep.2002.33683 (2002). 12029633

[b6] Hori T. *et al.* Covalent modification of all members of human cullin family proteins by NEDD8. Oncogene 18, 6829–6834, 10.1038/sj.onc.1203093 (1999). 10597293

[b7] Sharma P. & Nag A. CUL4A ubiquitin ligase: a promising drug target for cancer and other human diseases. Open Biol 4, 130217, 10.1098/rsob.130217 (2014). 24522884 PMC3938054

[b8] Shinomiya T. *et al.* Comparative genomic hybridization of squamous cell carcinoma of the esophagus: the possible involvement of the DPI gene in the 13q34 amplicon. Genes Chromosomes Cancer 24, 337–344 (1999). 10092132

[b9] Dohna M. *et al.* Adrenocortical carcinoma is characterized by a high frequency of chromosomal gains and high-level amplifications. Genes Chromosomes Cancer 28, 145–152 (2000). 10824999

[b10] Birner P. *et al.* Human homologue for Caenorhabditis elegans CUL-4 protein overexpression is associated with malignant potential of epithelial ovarian tumours and poor outcome in carcinoma. J Clin Pathol 65, 507–511, 10.1136/jclinpath-2011-200463 (2012). 22447918

[b11] Xu Y., Wang Y., Ma G., Wang Q. & Wei G. CUL4A is overexpressed in human pituitary adenomas and regulates pituitary tumor cell proliferation. J Neurooncol 116, 625–632, 10.1007/s11060-013-1349-2 (2014). 24420924

[b12] Chen L. C. *et al.* The human homologue for the Caenorhabditis elegans cul-4 gene is amplified and overexpressed in primary breast cancers. Cancer Res 58, 3677–3683 (1998). 9721878

[b13] Schindl M., Gnant M., Schoppmann S. F., Horvat R. & Birner P. Overexpression of the human homologue for Caenorhabditis elegans cul-4 gene is associated with poor outcome in node-negative breast cancer. Anticancer Res 27, 949–952 (2007). 17465225

[b14] Wang Y. *et al.* CUL4A overexpression enhances lung tumor growth and sensitizes lung cancer cells to Erlotinib via transcriptional regulation of EGFR. Mol Cancer 13, 252, 10.1186/1476-4598-13-252 (2014). 25413624 PMC4246448

[b15] Wang Y. *et al.* CUL4A induces epithelial-mesenchymal transition and promotes cancer metastasis by regulating ZEB1 expression. Cancer Res 74, 520–531, 10.1158/0008-5472.CAN-13-2182 (2014). 24305877 PMC3934357

[b16] Wang Y. *et al.* Involvement of CUL4A in regulation of multidrug resistance to P-gp substrate drugs in breast cancer cells. Molecules 19, 159–176, 10.3390/molecules19010159 (2013). 24368600 PMC6271407

[b17] Sanyal A. J., Yoon S. K. & Lencioni R. The etiology of hepatocellular carcinoma and consequences for treatment. Oncologist 15, Suppl 4, 14–22, 10.1634/theoncologist.2010-S4-14 (2010). 21115577

[b18] Yim H. J. & Lok A. S. Natural history of chronic hepatitis B virus infection: what we knew in 1981 and what we know in 2005. Hepatology 43, S173–181, 10.1002/hep.20956 (2006). 16447285

[b19] van Zijl F. *et al.* Epithelial-mesenchymal transition in hepatocellular carcinoma. Future Oncol 5, 1169–1179, 10.2217/fon.09.91 (2009). 19852728 PMC2963061

[b20] Arzumanyan A., Reis H. M. & Feitelson M. A. Pathogenic mechanisms in HBV- and HCV-associated hepatocellular carcinoma. Nat Rev Cancer 13, 123–135, 10.1038/nrc3449 (2013). 23344543

[b21] Hung M. S. *et al.* Cul4A is an oncogene in malignant pleural mesothelioma. J Cell Mol Med 15, 350–358, 10.1111/j.1582-4934.2009.00971.x (2011). 19929949 PMC3355981

[b22] Jiang L., Rong R., Sheikh M. S. & Huang Y. Cullin-4A.DNA damage-binding protein 1 E3 ligase complex targets tumor suppressor RASSF1A for degradation during mitosis. J Biol Chem 286, 6971–6978, 10.1074/jbc.M110.186494 (2011). 21205828 PMC3044953

[b23] Rosenblatt J., Gu Y. & Morgan D. O. Human cyclin-dependent kinase 2 is activated during the S and G2 phases of the cell cycle and associates with cyclin A. Proc Natl Acad Sci USA 89, 2824–2828 (1992). 1532660 10.1073/pnas.89.7.2824 PMC48755

[b24] Pines J. & Hunter T. Human cyclins A and B1 are differentially located in the cell and undergo cell cycle-dependent nuclear transport. J Cell Biol 115, 1–17 (1991). 1717476 10.1083/jcb.115.1.1 PMC2289910

[b25] Park T. J. *et al.* TIS21 negatively regulates hepatocarcinogenesis by disruption of cyclin B1-Forkhead box M1 regulation loop. Hepatology 47, 1533–1543, 10.1002/hep.22212 (2008). 18393292

[b26] Wang L. H., Huang W., Lai M. D. & Su I. J. Aberrant cyclin A expression and centrosome overduplication induced by hepatitis B virus pre-S2 mutants and its implication in hepatocarcinogenesis. Carcinogenesis 33, 466–472, 10.1093/carcin/bgr296 (2012). 22159224

[b27] Ogunwobi O. O. & Liu C. Therapeutic and prognostic importance of epithelial-mesenchymal transition in liver cancers: insights from experimental models. Crit Rev Oncol Hematol 83, 319–328, 10.1016/j.critrevonc.2011.11.007 (2012). 22178416

[b28] Thompson E. W., Newgreen D. F. & Tarin D. Carcinoma invasion and metastasis: a role for epithelial-mesenchymal transition? Cancer Res 65, 5991–5995; discussion 5995, 10.1158/0008-5472.CAN-05-0616 (2005). 16024595

[b29] Edmondson H. A. & Steiner P. E. Primary carcinoma of the liver: a study of 100 cases among 48,900 necropsies. Cancer 7, 462–503 (1954). 13160935 10.1002/1097-0142(195405)7:3<462::aid-cncr2820070308>3.0.co;2-e

[b30] Han C. P. *et al.* Scoring of p16(INK4a) immunohistochemistry based on independent nuclear staining alone can sufficiently distinguish between endocervical and endometrial adenocarcinomas in a tissue microarray study. Mod Pathol 22, 797–806, 10.1038/modpathol.2009.31 (2009). 19347018

[b31] Kamoi S., AlJuboury M. I., Akin M. R. & Silverberg S. G. Immunohistochemical staining in the distinction between primary endometrial and endocervical adenocarcinomas: another viewpoint. Int J Gynecol Pathol 21, 217–223 (2002). 12068166 10.1097/00004347-200207000-00003

[b32] Han C. P. *et al.* Nuclear Receptor Interaction Protein (NRIP) expression assay using human tissue microarray and immunohistochemistry technology confirming nuclear localization. J Exp Clin Cancer Res 27, 25, 10.1186/1756-9966-27-25 (2008). 18673574 PMC2683569

[b33] Yan W. *et al.* Tim-3 fosters HCC development by enhancing TGF-beta-mediated alternative activation of macrophages. Gut 64, 1593–1604, 10.1136/gutjnl-2014-307671 (2015). 25608525

[b34] Li X. *et al.* Cullin 4B protein ubiquitin ligase targets peroxiredoxin III for degradation. J Biol Chem 286, 32344–32354, 10.1074/jbc.M111.249003 (2011). 21795677 PMC3173229

[b35] Du J. *et al.* Hepatitis B virus core protein inhibits TRAIL-induced apoptosis of hepatocytes by blocking DR5 expression. Cell Death Differ 16, 219–229, 10.1038/cdd.2008.144 (2009). 18927587

[b36] Liang X. *et al.* Hepatitis B virus sensitizes hepatocytes to TRAIL-induced apoptosis through Bax. J Immunol 178, 503–510 (2007). 17182590 10.4049/jimmunol.178.1.503

[b37] Luan F. *et al.* Hepatitis B virus protein preS2 potentially promotes HCC development via its transcriptional activation of hTERT. Gut 58, 1528–1537, 10.1136/gut.2008.174029 (2009). 19651630

[b38] Gai X. *et al.* Hepatitis B virus core protein enhances human telomerase reverse transcriptase expression and hepatocellular carcinoma cell proliferation in a c-Ets2-dependent manner. Int J Biochem Cell Biol 45, 1174–1185, 10.1016/j.biocel.2013.03.015 (2013). 23542016

